# Analysis of Different Routes of Hysterectomy Based on a Prospective Algorithm and Their Complications in a Tertiary Care Institute

**DOI:** 10.1155/2022/6034113

**Published:** 2022-09-15

**Authors:** Subrat Panda, Ananya Das, Rituparna Das, Nalini Sharma, Wansalan Shullai, Vinayak Jante, Anusuya Sharma, Kaushiki Singh, Prateeti Baruah, Ruksana Makakmayum

**Affiliations:** NEIGRIHMS, Shillong, India

## Abstract

**Introduction:**

Hysterectomy is the most common gynaecological operation worldwide. The objective of the study is to analyze the various routes of hysterectomy and its complications when the decision of route is based on using a prospective algorithm tree. *Methodology*. It is an observational study to analyze the route of hysterectomy based on using a prospective algorithm. The decision tree is based on pelvic pathology, uterine size, vaginal access, pelvic adhesion, competency of the surgeon, choice of the patient, and complication of different routes of hysterectomy. Data were collected from preoperative, intraoperative, and postoperative records. Demographic factors, indications, routes of hysterectomy, and complications were recorded and analyzed by using SPSS software version 22. *Observation*. Among the malignant or suspected malignant pathology groups, TAH was performed in 89 cases and TLH was performed in 3 cases. Among the benign disease groups, VH was performed in 137(38.2%) cases, TAH was performed in 118(32.9%) cases, and TLH was performed in 104 (28.9%) cases. Operative time and a number of blood transfusions were significantly less with VH (*p* value < 0.0001 and 0.004) compared to abdominal and total laparoscopic hysterectomy. Postoperative complication such as fever was more with abdominal hysterectomy (<i>*p*-</i>value<0.00001) compared to VH and TLH. Vaginal discharge was more with VH and TLH compared to TAH (*p* value −0.004) and wound infection was more in the abdominal route (*p* value 0.001).

**Conclusion:**

The abdominal route was the route of choice for surgery in malignancy or suspected malignant pathology. In benign pathology, VH was the most common and preferable route of surgery. Complications were found to be minimal with vaginal hysterectomy.

## 1. Introduction

A substantial number of women undergo hysterectomy annually and 70% of hysterectomies are performed for benign indications, including leiomyoma, adenomyosis, and uterine prolapse [1]. To ensure that each patient receives the best possible care at reasonable costs, physicians must closely analyze the recent data comparing surgical approaches to hysterectomies [2]. Abdominal hysterectomy is associated with less favorable medical outcomes, thereby evidence supports its use only when documented pathologic conditions preclude the use and efficiency of the vaginal route [3 to 6]. Many times surgeons neglect to adopt evidence-based formal practice guidelines for hysterectomy and often choose a route of hysterectomy based on their personal preference. Historically, abdominal hysterectomy is accepted to be appropriate for more serious diseases that necessitate this approach. However, traditional teaching predisposes surgeons to select the abdominal route despite pathologic indications. Laparoscopic hysterectomy is reported to have lower postoperative morbidity, improved quality of life, shorter hospital stay, and less blood loss when compared to laparotomy [7, 8]. A laparoscopic approach may not be feasible in patients with a history of multiple abdominal surgeries, dense pelvic and bowel adhesions, and large fibroids, wherein laparotomy takes the upper hand. Gynaecologic surgeons should be vigilant for these indicators when examining patients. Gynaecologic surgeons must clearly decide the route of hysterectomy based on the patient's safety and also a wise use of health care expenditure specifically pertaining to hospital stay and cost for managing complications. Developing clinical guidelines based on accurate physical findings is the first step in ensuring that women will undergo the most appropriate route of hysterectomy that is cost-effective and meets the standard of quality care [7 to 9]. According to ACOG guideline 2017, the vaginal route is the preferred route for hysterectomy whenever it is feasible.

Our study was an observational study conducted to analyze the routes of hysterectomy and its complications in a tertiary care teaching hospital when the types of hysterectomy are based on using a prospective algorithm and decision tree based on the indication of hysterectomy, uterine size, vaginal access, pelvic adhesion, and competency of the surgeon and choice of women undergoing hysterectomy.

## 2. Materials and Methods

It was a prospective observational cross-sectional, descriptive study conducted in the Department of Obstetrics and Gynaecology in NEIGRIHMS to analyze the route of hysterectomy and their intraoperative and postoperative complications. We followed the optimal surgical route for hysterectomy using a prospective algorithm tree.

The algorithm tree included indication (benign/malignancy/suspected malignancy). In the malignancy and suspected malignancy group, the route of choice was the abdominal route. Among the benign pathology, the route of hysterectomy was decided according to the size of the uterus. Vaginal hysterectomy was chosen for a uterine size up to 14 weeks, 14 to 26 weeks size uterus was treated by laparoscopic hysterectomy, and uterine size above 26 weeks was managed by abdominal hysterectomy.

Pelvic and abdominal adhesion was assessed clinically and in some cases with the help of MRI when it was difficult to determine the extent of adhesion clinically. Vaginal accessibility and descent were also assessed. When adhesion was present, even with the size of the uterus less than 14 weeks laparoscopic route or abdominal route was preferred.

In our study, competency of the surgeon and the choice of the women pertaining to the route of surgery were taken into consideration, as the last decision-making factor. The competency of the surgeon in our study was a factor to decide the route of hysterectomy because ours is a postgraduate teaching institute and have junior to senior faculty and even a senior residency program is running as a part of training.

This study was conducted in NEIGRIHMS from June 2018 to May 2020. One senior resident doctor was assigned to collect the data about the hysterectomies performed during this period. Data were collected every week. Data included the preoperative, intraoperative, and postoperative case records of patients. Age, parity, BMI, the indication of hysterectomy, pathology, comorbidities, and size of the uterus were noted from the preoperative records. Type of anesthesia, duration of operation, blood loss during operation, and requirement for blood transfusion or any other complication during surgery were notified in the intraoperative record. From the postoperative records, any complication and duration of hospital stay were recorded. Readmission of operated patients and long-term postoperative complaints were also documented. The intraoperative and postoperative complications were analyzed among three routes of hysterectomy (TAH, VH, and TLH).

In cases of TLH, we also compared the intraoperative and postoperative complications between endosuturing and vaginal suturing of the vault. In our institute, we performed a total hysterectomy laparoscopically, extracted the specimen vaginally, and sutured the vault vaginally. In some cases, after extracting the specimen vaginally we did endosuturing of the vault. All the demographic factors were recorded. Intraoperative and postoperative complications were analyzed according to the route of hysterectomy by using SPSS software version 22. The study was conducted after getting permission from the Medical Superintendent's Office and Medical Record Department.

## 3. Results and Observations

**Figure 1:**
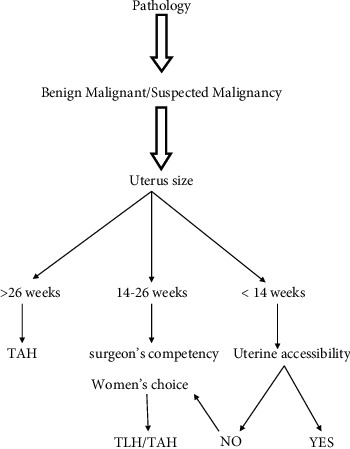
Algorithm for hystrectomy.

We had performed 451 hysterectomies in our institute for 2 years period from June 2018 to May 2020, out of which 207(45.8%) were transabdominal, 107(23.7%) were total laparoscopic hysterectomies, and 137(30.3%) were vaginal hysterectomies (Table1). In our study we had 92 cases having malignancy or suspected malignancy, which were operated abdominally except 3 cases of carcinoma endometrium which were operated laparoscopically. Among the benign pathology group, 118(32.9%) had an abdominal hysterectomy, 104(28.9%) had a laparoscopic hysterectomy, and 137(38.2%) had a vaginal hysterectomy. Out of all vaginal hysterectomies, 20 were Ward Mayo's operation for prolapsed uterus and the rest were nondescent hysterectomies (Figure 1). Among the benign conditions, especially leiomyoma of the uterus and DUB were the common pathology and the route of surgery was based on the size of the uterus and vaginal accessibility and adhesion. In the case of ovarian cysts, if any factors suggested suspicion of malignancy, the abdominal route was preferred according to RMI Score. 36 cases had an abdominal hysterectomy, and in 6 cases, TLH was performed. In cases of endometriosis, all hysterectomies were performed either abdominally or laparoscopically. Among the nulliparous women, 15 cases were performed abdominally, 12 cases vaginally, and 6 laparoscopically (Table 2). No significant difference was seen when groups were compared according to demographic factors. Among the women with uterus, less than 14 weeks in size, in 117(45.7%) cases, hysterectomy was performed by abdominal route, 107 (41.7%) cases were performed vaginally, and 32 (12.5%) cases were performed laparoscopically (*p*-value <0.0001). In 14 to 26 weeks size uterus, 57 were performed abdominally, 62 laparoscopically, and 30 cases vaginally (*p*-value < 0.006). In more than 26weeks size uterus, 33 cases were performed abdominally and 13 laparoscopically (*p*-value <0.0001) (Table 3). Comorbidities associated with hysterectomy cases were hypertension, anemia, thyroid disorder, cardiovascular disease, and diabetes mellitus. Most of the vaginal and abdominal hysterectomies were performed with regional anesthesia, and all laparoscopic hysterectomies were performed with general anesthesia. There was no significant difference in operative blood loss of more than 1000 ml (*p*-value 0.125) according to the route of hysterectomy. Blood transfusion was less with the vaginal hysterectomy group compared to the abdominal and laparoscopy route (*p* value 0.0032). Bilateral salpingectomy was performed in a fewer cases of vaginal hysterectomy compared to the abdominal and laparoscopic route (Table 4). The incidence of ureteric, urinary bladder, and bowel injury was not statistically significant among the three routes of hysterectomy. There was a significant difference in the operative time of less than 30 minutes in three routes of hysterectomies (abdominal l5.7%, laparoscopy 1.8%, and vaginal 16.8% with *p* value < 0.001). Laparoscopic hysterectomy took a long time of 1 to 3 hours in 85% of cases, whereas 68.5% of abdominal and 82.3% of vaginal hysterectomies were performed within 1 hour. 6 laparoscopic hysterectomies had to be converted to abdominal hysterectomy. The incidence of postoperative fever was significantly higher in the abdominal route (52.1%, 38.75%, and 28.4%) compared to laparoscopic and vaginal routes of hysterectomy. The mean number of hospital stay in abdominal hysterectomy was 9 days, whereas it was 4 days for the laparoscopic and 3 days for the vaginal hysterectomy group. Vaginal discharge was less with abdominal hysterectomy compared to laparoscopic and vaginal hysterectomy cases (<i>*p* </i>value0.004). In postoperative complications, vesico vaginal fistula was seen in 2 cases of abdominal hysterectomies and 1 case of laparoscopic hysterectomy. A mild degree of peritonitis was also seen in one case of vaginal hysterectomy. The incidence of urinary tract infection in 3 routes was nonsignificant (*p* value −0.2509). Wound infection was more in abdominal hysterectomy compared to total laparoscopic hysterectomy (14.4% Vs 7.1%; *p* value 0.001). A burst abdomen was seen in 2 cases of abdominal hysterectomy. Among the laparoscopic hysterectomy, we did endosuturing of the vault in 29 cases only. Fever, vaginal discharge, and UTI were less in the endosuturing group (*p* values 0.0001, 0.0001, and 0.0001) (Table 5).

## 4. Discussion

Hysterectomy is widely used for treating a variety of gynecologic conditions. Most hysterectomies are elective and are performed to treat benign indications. The lifetime risk of hysterectomy for a woman in the United States is 45% [10] Hysterectomy remains the second most commonly performed surgical procedure for women of reproductive age, second only to cesarean section [11]. It is a treatment option for many benign and malignant conditions but not free of associated morbidity and mortality [12]. In our study, there is no significant difference in demographic value among the three groups of hysterectomies. Most of the women belonged to the 40 to 50 years age group. A similar age group was observed in other studies conducted by Sivapragasam et al. [13]. In our study, the most common indication was dysfunctional uterine bleeding followed by leiomyoma. A study conducted by Prasad et al. showed a 59.4% incidence of fibroid uterus in hysterectomy patients, the next common indication was abnormal uterine bleeding (23.3%) [14]. A study conducted by Sridevi et al. showed they had 16% hysterectomies due to prolapse of the uterus, but in our study, it was only (4.4%]. Prolapse of the uterus is very rare in our region. Most cases of U to V prolapse were referral cases from surrounding areas.

Many studies have compared surgical approaches and complications to determine which method is best for the patient. Abdominal hysterectomy is found to be inferior to vaginal hysterectomy and laparoscopic hysterectomy [15]. In our study postoperative fever was found more in abdominal hysterectomies. Higher blood transfusion in abdominal hysterectomy can be explained by the fact that complicated cases including malignancy were performed by the abdominal route. We had 207 (45.8%) cases of abdominal hysterectomy, 107(23.7%) cases of total laparoscopic hysterectomy, and 137(30.3%) cases of vaginal hysterectomy. In our study, all malignant and ovarian masses were operated on by the abdominal route, and cases of endometriosis were operated abdominally and laparoscopically because of adhesion and as the surgery required complete removal of all endometriotic tissue including deeply infiltrating endometriosis (DIE).

The surgical approach of hysterectomy is the most important factor responsible for post-operative complications. The mean hospital stay for abdominal hysterectomy was 9 days, 4 days for laparoscopic cases and 3 days for vaginal hysterectomy cases. The average number of hospital stays were 5.4 days in TLH, 6.3 days, and 7.4 days for abdominal hysterectomy and vaginal hysterectomy in a study by Hyo-Shin Kim et al. [16] In the same study, endoscopic surgery was converted to open surgery in four cases (0.5%). In our study, 5.7% of cases of laparoscopic hysterectomy were converted to abdominal hysterectomy. This finding may be due to complications and the wrong choice of route by the surgeon. Postoperative fever was more in abdominal hysterectomy compared to vaginal and laparoscopic groups (*p*-value-0.00001). In a meta-analysis by Johnson et al. laparoscopy was associated with fewer infections (OR 0.32) and fewer episodes of fever (OR 0.65), compared with abdominal hysterectomy. Similar findings were noted comparing vaginal and abdominal hysterectomies [17]. Urinary tract infection was similar in the abdominal, laparoscopic, and vaginal groups (*p* value −0.56) but the vaginal discharge was more in the vaginal and laparoscopic route (*p* value −0.004). In our study, in laparoscopic hysterectomy, most of the cases had vault closure vaginally. Wound infection and burst abdomen were more in the abdominal route compared to the laparoscopic route in a statistically significant way (*p* value −0.001). In the study in Finland in 1996 by Makinen et al., infections of wounds, intra-abdominal structures, vagina, urinary tract, and fever of unknown origin, were the most frequent complications, with an incidence of 10.5%, 13%, and 9% in the abdominal, vaginal, and laparoscopic hysterectomy groups, respectively [18]. In our study, no statistically significant difference of intraoperative complications such as urinary bladder, ureteric, and bowel injuries in three modes of hysterectomy were found. A study by Hyo-Shin Kim et al. had similar rates of urinary and bowel injury [16]. We did not have a single case of vaginal dehiscence in the three groups of hysterectomies. 82.3% vaginal hysterectomy was performed within 1 hour but most of the abdominal (88.4%) and laparoscopic hysterectomy (85%) were performed within an hour to 3 hours. Cochrane review in 2015, showed that the operation time of VH was significantly faster than that of laparoscopic hysterectomy. Compared with the abdominal route, the beneficial effects of VH included fewer febrile episodes or unspecified infections, a shorter duration of hospital stays, lower intraoperative blood loss, and fewer wound complications [19]. Operative time was shorter in TAH than in LH (2.22 ± 0.93hours vs 2.43 ± 0.94 hours, respectively) but it was not statistically significant. [20] In our study, salpingectomy was performed at 57.8% in abdominal hysterectomy, 57% laparoscopic route, and 27% vaginal route (*p* value 0.0001). A study by Natalie De Cure and Stephen showed VH had 15.9% salpingectomy, AH had 66.9%, and LH had 84.5% of salpingectomy. [21] We had similar rates of blood transfusion in the abdominal (14%) and laparoscopic (14.4%) groups but quite less in the vaginal hysterectomy group (2.5%). Radhika Ganesh et al. in their study had more blood loss in the vaginal hysterectomy group and least in the laparoscopic group [21] In our case blood loss was less with vaginal hysterectomy, which can be explained by the use of bipolar clamp for the surgery. Among the TLH group, we had only 29 cases of endosuturing of the vault and in other cases, we closed the vault transvaginally. Postoperative fever, UTI, and vaginal discharge were more in transvaginal closure of the vault. A study by Kanupriya Singh et al. showed only 28.5% of patients.

We had various postoperative complications in the laparoscopic route of vault closure as compared to 88.5% in the vaginal route of vault closure [22]. In our study, more operative time and complication were with abdominal hysterectomy, followed by a total laparoscopic hysterectomy, and then with vaginal hysterectomy. In our study, in most of the malignancy cases and suspected malignancy we did abdominal surgery. In the case of cervical cancer, Ramirez PT et al. provided evidence of poorer outcomes for minimally invasive radical hysterectomy than abdominal radical hysterectomy, among women with early-stage cervical cancer [23]. In our study we did abdominal hysterectomy for all the cervical and ovarian maliganancy cases.

In cases of benign conditions, ACOG 2017 recommends vaginal hysterectomy as the approach of choice whenever feasible. Evidence demonstrates that it is associated with better outcomes when compared with other approaches to hysterectomy. Laparoscopic hysterectomy is a preferable alternative to open abdominal hysterectomy for those patients in whom a vaginal hysterectomy is not indicated or feasible. Selection of the route of hysterectomy for benign causes can be influenced by the size and shape of the vagina and uterus, accessibility of the uterus (e.g., descents and pelvic adhesions), extent of extrauterine disease, the need for concurrent procedures, surgeon training and experience, average case volume, available hospital technology, devices, and support whether the case is emergent or scheduled, and preference of the informed patient. Although VH generally has the advantage of less intraoperative blood loss, fewer postoperative complications, better cosmetic results, and quicker recovery, it is not often the choice when a large-sized uterus is encountered. [24].

Concomitant adnexal surgery increased the likelihood of undergoing abdominal surgery rather than vaginal [25]. We had only 10% adnexectomy in the vaginal route. VH is the route of choice in obese patients and LH should be selected over AH in obese patients for whom a vaginal approach is not feasible [26]. In our study, there was no significant difference in BMI for the three modes of hysterectomies.

Since ours is a postgraduate and undergraduate teaching institution, we follow the principle of competency in abdominal, vaginal, and laparoscopic hysterectomy in chronology. We follow the optimal surgical route for hysterectomy using a prospective algorithm and decision tree based on the indication of hysterectomy (benign/suspected benign/malignancy) uterine size, abdominopelvic adhesion, and vaginal access. Lastly, included factors for the route of hysterectomy were the competency of the surgeon and the choice of the women undergoing hysterectomy. In our study based on a prospective algorithm, 38.2% had a vaginal hysterectomy, 32.9% had an abdominal hysterectomy, and 28.9% had a total laparoscopic hysterectomy. For malignancy, 89 cases had an abdominal hysterectomy and 3 had a total laparoscopic hysterectomy (Figure 1).

## 5. Emerging Trends in Vaginal Hysterectomy

Nowadays, vaginal natural orifices transluminal endoscopic surgery (vNOTES) hysterectomy provides favorable outcomes compared to conventional LH considering the shorter operation time, hospitalization, and lower 24th-hour VAS score [27]. The duration of surgery was significantly shorter in the vNOTES hysterectomy group (79.56 ± 32.54 min) compared to the TLH group (120.67 ± 38.35 min) (*p*: <.001). Also, postoperative hospital stays were significantly shorter in favor of the vNOTES hysterectomy group (44 ± 16.47 h) compared to the TLH group (57.86 ± 21.31 h) (*p* : 0.002). These results indicate that vNOTES hysterectomy can be a promising approach for treating a variety of different uterine pathologies and also in cases of large uterus size with adhesion. Furthermore, it can also be an alternative to TLH [28].

## 6. Conclusion

The abdominal route is the preferred route for hysterectomy in gynaecological malignancies and suspected malignancies. Even though the optimal surgical route for hysterectomy in benign pathology remains vaginal hysterectomy. A prospective algorithm and decision tree for the indication of hysterectomy, uterine size, vaginal access, and abdominopelvic adhesion are used to decide the best route. The competency of the surgeon and the wish of the women about the route of hysterectomy is an important factor. Vaginal hysterectomy has minimal complications than the laparoscopic and abdominal route and is hence preferred.

## Figures and Tables

**Table 1:** Different types of hysterectomy and indications

	TAH	TLH	VH	Total
Total number of procedures	207 (45.8%)	107 (23.7%)	137 (30.3%)	451
Indications				
Malignancy or suspected malignancy	**89**	**3**	**0**	**92**
Benign	118 (32.9%)	104 (28.9%)	137 (38.2%)	359
Uterine fibroid	47 (39.9)	37 (31.3)	34 (28.7%)	25.27%
DUB	48 (30.3)	47 (29.7)	63 (40%)	33.9%
Utero-vaginal prolapse	0	0	20	4.4%
Ca ovary	14	0	0	3.1%
Ovarian tumors and cysts	36 (85.7%)	6 (14.3%)	0	7.7%
Ca endometrium	17 (85%)	3 (15%)	0	4.4%
Endometrial hyperplasia with atypia	2 (20%)	0	8 (80%)	2.2%
Molar pregnancy	2	0	0	0.4%
GTN	2	0	0	0.4%
Ca-cervix	17	0	0	3.7%
Adenomyosis	5 (23.8%)	4 (14.2%)	12 (57.1%)	4.6%
Endometriosis	14 (58.3%)	10 (41.7%)	0	5.3%
Leiomyosarcoma	3	0	0	0.4%

**Table 2:** Different types of hysterectomy and demographic features.

	TAH	TLH	VH	*p* value
Age (in years)				
40 to 50	137 (66.1%)	52 (48.5%	84 (61.3%)	0.01
50 to 60	52 (25.1%)	43 (40.1%)	43 (31.3%	0.022
60 to 75	18 (8.6%)	12 (11.2%)	10 (7.29%)	0.56
Mean BMI	23.5	24.1	24.6	0.999
Parity				
Nulliparous	15	6	12	0.643
P1 to P5	102	65	66	0.09
>P5	90	36	5	

**Table 3:** Different types of hysterectomy and preoperative findings.

	TAH	TLH	VH	*p* value
Size of uterus less than 14 weeks	117 (45.7%)	32 (12.5%)	107 (41.7%)	<0.0001
14 to 26 weeks	57 (38.2)	62 (41.6%)	30 (20.13)	<0.006
More than 26 weeks	33 (71.7%)	13 (28.2%)		<0.001
Comorbidities hypertension	17 (8.2%)	10 (9.3%)	12 (8.7%)	**0.473**
Diabetes	15 (7.2%)	10 (9.3%)	12 (8.7%)	**0.434**
Cardiac diseases	10			
Thyroid disorder	30 (13.8%)	18 (16.8%)	20 (14.5%)	**0.238**
Anemia	84 (40.5%)	40 (37.3%)	54 (39.4%)	**0.027**

**Table 4:** Different types of hysterectomy and intraoperative findings.

	TAH	TLH	VH	*p* value
Anaesthesia General	57	107	20	
Regional	150		117	
Blood loss>1000 ML	25 (12%)	9 (8%)	4 (5.8%)	0.1245
Blood transfusion	30 (14.4%)	15 (14%)	4 (2.9%)	0.0032
B/L salpingectomy	137 (57.8%)	61(57%)	31(27%)	0.004
BSO	57 (71.25)	15(18.75%)	8(10%)	0.011
BSO + omentectomy + RPLND + BPLND	24	0	0	
BSO + mesenteric cyst excision	1	0	0	
Laparoscopic cholecystectomy	0	6	0	
Pelvic floor repair	0	0	10	
Injury				
Ureteric	3 (1.4%)	1 (0.9%)	0	
Urinary bladder	5 (2.4%)	3 (2.8%)	2 (1.4%)	0.5902
Bowel	3 (1.4%)	1 (0.9%)	0	0.6212
Conversion to other routes		6 converted to TAH		
Operative time				
Less than 30 mins.	12 (5.7%)	2 (1.8%)	23 (16.7%)	<0.001
30 to 60 mins.	130 (62.8%)	30 (28.03%)	90 (65.6%)	<0.0001
1 to 3 hours	53 (25.6%)	60 (56.07%)	24 (17.5%)	<0.0001
3 to 5 hours	12	15		

**Table 5:** Different types of hysterectomy and postoperative events.

Postop events	TAH	TLH	VH	*p* value
Fever	108 (52.1%)	27 (33.75%)	39 (28.4%)	<0.00001
Mean hospital stay	9	4	4	
VVF	2 (0.9%)	1 (0.9%)	0	
Peritonitis	0	0	1 (2.7%)	
Vaginal discharge	40 (19.3%)	30 (37.5%)	48 (35.03%)	0.004
UTI	52 (25.1%)	23 (28.7%)	38 (27.7%)	0.53
Wound infection	30 (14.4%)	3 (3.7%)	0	0.001
Burst abdomen	2	0	0	

## Data Availability

The excel format of data used to support the findings of this study to find the right approach to hysterectomy are available from the corresponding author upon request, Dr. Subrat Panda, email ID: subrtpanda@gmail.com.

## References

[1] Whiteman M. K., Hillis S. D., Jamieson D. J. (2008). Inpatient hysterectomy surveillance inthe United States, 2000–2004. *American Journal of Obstetrics and Gynecology*.

[2] Kovac S. R. (2014). Route of hysterectomy: an evidence-based approach. *Clinical Obstetrics and Gynecology*.

[3] Dicker R. C., Greenspan J. R., Strauss L. T. (1982). Complications of abdominal and vaginalhysterectomy among women of reproductive age in the United States. TheCollaborative Review of Sterilization. *American Journal of Obstetrics and Gynecology*.

[4] Kovac S. R. (1995). Guidelines to determine the route of hysterectomy. *Obstetrics & Gynecology*.

[5] Richardson R. E., Bournas N., Magos A. (1995). Is laparoscopic hysterectomy a waste of time?. *The Lancet*.

[6] Querleu D., Cosson M., Paramentier D., Debodinance P. (1993). The impact of laparoscopicsurgery on vaginal hysterectomy. *Gynecol Endosc*.

[7] Kluivers K. B., Johnson N. P., Chien P., Vierhout M. E., Bongers M., Mol B. W. (2008). Comparison of laparoscopic and abdominalhysterectomy in terms of quality of life: a systematic review. *European Journal of Obstetrics & Gynecology and Reproductive Biology*.

[8] Walsh C. A., Walsh S. R., Tang T. Y., Slack M. (2009). Total abdominal hysterectomy versus totallaparoscopic hysterectomy for benign disease: a meta-analysis. *European Journal of Obstetrics & Gynecology and Reproductive Biology*.

[9] Kovac S. R. (2000). Decision-directed hysterectomy: a possible approach to improve medicaland economic outcomes. *International Journal of Gynecology & Obstetrics*.

[10] Merrill R. M. (2008). Hysterectomy surveillance in the United States-1997 through 2005. *Medical Science Monitor*.

[11] Buie V. C., Owings M. F., DeFrances C. J., Golosinskly A. (2010). *National Hospital DischargeSurvey: 2006 Summary*.

[12] Wu J. M., Wechter M. E., Geller E. J., Nguyen T. V., Visco A. G. (2007). Hysterectomy rates in the United States, 2003. *Obstetrics & Gynecology*.

[13] Sivapragasam V., Rengaswamy C. K., Patil A. B. (2018). An audit of hysterectomies: indications, complications, clinic pathological analysis of hysterectomy specimens in a tertiarycare center. *International Journal of Reproduction, Contraception, Obstetrics and Gynecology*.

[14] Prasad D. R., Nair N. V. (2018). Retrospective analysis of elective hysterectomy cases in a tertiary care centre. *IInternational Journal of Reproduction, Contraception, Obstetrics and Gynecology*.

[15] Aarts J. W. M., Nieboer T. E., Johnson N. (2015). Surgical approach to hysterectomy for benign gynaecological disease. *Cochrane Database of Systematic Reviews*.

[16] Kim H. S., Koo Y. J., Lee D. H. (2020). Clinical outcomes of hysterectomy for benign diseases in the female genital tract: 6 years’ experience in a single institute. *Yeungnam university journal of medicine*.

[17] Johnson N., Barlow D., Lethaby A., Tavender E., Curr L., Garry R (2005). Methods of hysterectomy: systematic review and meta-analysis of randomised controlled trials. *BMJ*.

[18] Mäkinen T. B., Sjoberg J. (2013). Ten years of progress—improvedhysterectomy outcomes in Finland 1996–2006: a longitudinal observational study. *Juha*.

[19] Nieboer T. E., Johnson N., Lethaby A., Tavender E., Curr E., Garry R. (2009). Surgicalapproach to hysterectomy for benign gynecological disease. *Cochrane Database of Systematic Reviews*.

[20] Aboulfotouh M. E. (2020). Fouad Chaalan & Abdelbaset Fakhry Laparoscopichysterectomy versus total abdominal hysterectomy: a retrospective study at a tertiaryhospital. *Gynecological Surgery*.

[21] Radhika Ganesh Y., Gowthami D., Venkateshwar Reddy J., Rani (2017). MeritsAnd demerits of different routes of hysterectomy for gynecological ConditionsWithout uterine. *DescentJournal of Dental and Medical Sciences*.

[22] Singh K., Shah B. (2011). Vaginal vault closure techniques in totallaparoscopic hysterectomy: a comparison between laparoscopic route vault suturingand vaginal route suturing. *National Journal of Community Medicine*.

[23] Ramirez P. T., Frumovitz M., Pareja R. (2018). Minimallyinvasive versus abdominal radical hysterectomy for cervical cancer. *New England Journal of Medicine*.

[24] Sutton C. (1997). Hysterectomy: a historical perspective. *Baillière’s Clinical Obstetrics and Gynaecology*.

[25] Wong F. W. S., Lim D. C. E. (2013). Factors influencing the choice of hysterectomy approach for the management of fibroid uterus. *Gynecology and Minimally Invasive Therapy*.

[26] Brezina P. R., Beste T. M., Nelson K. H., Nelson K. H. (2009). Does route ofHysterectomy affect outcome in obese and nonobese women?. *Journal of the Society of Laparoendoscopic Surgeons*.

[27] Kaya C., Yıldız Ş., Alay I. (2022). Comparison of surgical outcomes of total laparoscopic hysterectomy and vNOTES hysterectomy for undescended-enlarged uteri. *Journal of Investigative Surgery*.

[28] Kaya C., Alay I., Cengiz H., Yildiz G. O., Baghaki H. S., Yasar L. (2021). Comparison of hysterectomy cases performed via conventional laparoscopy or vaginally assisted natural orifice transluminal endoscopic surgery: a paired sample cross-sectional study. *Journal of Obstetrics and Gynaecology*.

